# Development of an *In Vitro* Assay for Detection of Drug-Induced Resuscitation-Promoting-Factor-Dependent Mycobacteria

**DOI:** 10.1128/AAC.00518-16

**Published:** 2016-09-23

**Authors:** Jessica Loraine, Feifei Pu, Obolbek Turapov, Galina V. Mukamolova

**Affiliations:** Department of Infection, Immunity, and Inflammation, University of Leicester, Leicester, United Kingdom

## Abstract

Tuberculosis is a major infectious disease that requires prolonged chemotherapy with a combination of four drugs. Here we present data suggesting that treatment of Mycobacterium tuberculosis, the causative agent of tuberculosis, and Mycobacterium smegmatis, a model organism widely used for the screening of antituberculosis agents, with first-line drugs resulted in the generation of substantial populations that could be recovered only by the addition of a culture supernatant from growing mycobacteria. These bacilli failed to grow in standard media, resulting in significant underestimation of the numbers of viable mycobacteria in treated samples. We generated M. smegmatis strains overexpressing M. tuberculosis resuscitation-promoting factors (Rpfs) and demonstrated their application for the detection of Rpf-dependent mycobacteria generated after drug exposure. Our data offer novel opportunities for validation of the sterilizing activity of antituberculosis agents.

## INTRODUCTION

Approximately one-third of the world's population is latently infected with Mycobacterium tuberculosis, the causative agent of human tuberculosis (TB) (1). M. tuberculosis bacilli can adapt to stressful conditions occurring at sites of host defense by reducing their metabolic activity and adopting a dormant state, often referred to as nonreplicating persistence (NRP) (2). Dormancy, or NRP, enables mycobacteria to survive environmental stresses and reduces the initiation of the immune response (3). Hence, dormancy can be defined as “a reversible state of low metabolic activity in which cells can exist for extended periods without division” (4).

Dormant bacilli, which are associated with latent infection, are believed to be less susceptible to antimicrobial treatment than actively growing bacilli due to physiological changes and therefore are of clinical significance in the eradication of TB (5). Nonreplicating M. tuberculosis bacilli generated in various *in vitro* models were more tolerant to rifampin and isoniazid than actively growing bacilli. These systems include the Wayne hypoxia model (2), starvation in phosphate buffer (6), a multiple-stress model (7), the persistence of a streptomycin-dependent strain (8), and a “nonculturability” model induced by potassium limitation (9). Thus, investigation of drug action on NRP bacteria and the development of *in vitro* models for the generation of such bacteria have been considered important for successful drug discovery programs. However, recent comparative studies on drug activities on NRP mycobacteria in *in vitro* systems showed significant differences (10). The presence of multiple mycobacterial populations with various physiological characteristics in NRP models may contribute to variability in the killing activity of drugs. Furthermore, the *in vivo* environment and exposure to stressful conditions may spawn heterogeneous mycobacterial populations with distinct cultivation requirements (11) and biochemical characteristics (12). Of particular interest are so-called resuscitation-promoting factor (Rpf)-dependent mycobacteria. Rpfs are a family of secreted peptidoglycan-remodeling enzymes, which are required for the regrowth of dormant bacteria (13). Rpf-dependent mycobacteria can be generated *in vitro* (14, 15), but most importantly, they are highly abundant in tuberculosis sputum (16) and can be recovered from infected lungs (11). Rpf-dependent M. tuberculosis bacilli from sputum have been shown to be more tolerant to rifampin (16), streptomycin, and isoniazid (17) than actively growing bacilli. Moreover, Hu and colleagues have recently demonstrated that Rpf-dependent M. tuberculosis bacilli were present in apparently sterile murine lungs in the Cornell model of latent tuberculosis infection and that the abundance of these bacilli correlated with tuberculosis reactivation (18). These observations suggest the necessity of monitoring the dynamics of Rpf-dependent M. tuberculosis during infection and treatment in order to predict chemotherapy outcomes for TB patients. Thus, there is strong demand for the development of simple assays to detect Rpf-dependent M. tuberculosis in clinical and experimental samples. The major challenge in Rpf dependency assays is posed by the high instability and unreliable activity of recombinant Rpf (19). Culture supernatants (SN) obtained from growing M. tuberculosis have been used as an alternative source for the detection of Rpf-dependent mycobacteria (16, 20, 21); however, this experimental setup is highly laborious and requires additional controls, preventing large-scale clinical studies and high-throughput drug testing. Moreover, other factors released into the culture supernatants of growing bacteria may contribute to the resuscitation of nonplatable (NP) bacteria and complicate the interpretation of results.

In this study, we demonstrate that treatment of mycobacterial cultures with first-line drugs results in the generation of bacteria that could be grown only in media supplemented with the culture supernatant. We have generated Mycobacterium smegmatis strains overexpressing M. tuberculosis Rpfs and have shown that these strains can be used for the detection of Rpf-dependent cells that arose after exposure to drugs. Our simple *in vitro* system can be used for assessing the efficacy of drugs and their propensity to generate Rpf-dependent mycobacteria.

## MATERIALS AND METHODS

### Bacteria and media.

M. smegmatis mc^2^155 or M. tuberculosis H37Rv was grown at 37**°**C. Bacteria were cultivated either in 7H9 Middlebrook medium (Becton, Dickinson and Company Ltd., Oxford, United Kingdom) supplemented with 0.2% (vol/vol) glycerol, 10% (vol/vol) oleic acid-albumin-dextrose-catalase (OADC), and 0.05% (wt/vol) Tween 80 or in Sauton's medium, unless otherwise stated. In some experiments, the OADC supplement was replaced with ADC; no significant difference in mycobacterial growth was found. Liquid cultures were shaken either at 100 rpm (M. tuberculosis) or at 200 rpm (M. smegmatis). For CFU assessment, mycobacteria were plated onto 7H10 Middlebrook (Becton, Dickinson and Company Ltd., Oxford, United Kingdom) agar. For maintenance of the pMind plasmid, kanamycin was added to a final concentration of 50 μg/ml; tetracycline (20 ng/ml) was added for induction of the expression of *rpf* genes.

### Assessment of viable counts.

CFU counts, using a standard liquid droplet method with serially diluted bacterial cultures, were performed in triplicate. Sealed agar plates were incubated at 37**°**C for as long as 1 week (M. smegmatis) or as long as 6 weeks (M. tuberculosis). Most probable number (MPN) counts were performed as described previously (16). Briefly, mycobacteria were serially diluted in 96-well (M. smegmatis) or 48-well (M. tuberculosis) microtiter plates containing growth broth with or without a culture supernatant prepared from actively growing M. tuberculosis cultures (16). For M. tuberculosis, 50 μl of mycobacteria was inoculated in 450 μl of 7H9 medium supplemented with 10% (vol/vol) OADC and 0.05% (vol/vol) Tween or in 450 μl of medium diluted with a culture supernatant (50% [vol/vol]). For M. smegmatis experiments, 20 μl of a bacterial suspension was inoculated in 180 μl of Sauton's medium with or without a culture supernatant. Four wells were used for each dilution and each medium. Plates were incubated for 2 weeks (M. smegmatis) or 12 weeks (M. tuberculosis) statically at 37°C before the number of positive wells was recorded and MPN calculated as described previously (16). Each sample was analyzed in triplicate; duplicate biological replicas were used per assay, and each independent assay was performed at least twice. The following terminology was introduced to describe mycobacteria in different physiological states: platable (CFU-producing) bacteria formed on solid media; “nonplatable” (NP) forms could grow only in liquid media; SN-dependent mycobacteria could be grown only in the presence of an Rpf-containing culture supernatant; Rpf-dependent mycobacteria required Rpf for growth (17). NP, SN-dependent, or Rpf-dependent mycobacteria were quantified in an MPN assay, and the resuscitation potential of mycobacterial populations was expressed as the resuscitation index (RI), calculated as log_10_(MPN) − log_10_(CFU).

### Growth studies.

Growth studies were carried out in supplemented 7H9 medium using an automated Bioscreen C MBR plate reader (Thermo Fisher Scientific) as described previously (22, 23). Cultures from the late-logarithmic phase were inoculated into supplemented 7H9 medium containing 50 μg/ml kanamycin and 20 ng/ml tetracycline to a final optical density (OD) of 0.1. Numbers of inoculated bacteria were confirmed by CFU counts. Plates were then incubated at 37°C with continuous shaking, and OD measurements were taken automatically every 2 h at 600 nm. Ten replicas were used per sample, and each experiment was performed a minimum of three times.

The growth rate (per hour) was calculated by plotting a log scale graph of linear growth and generating an exponential line showing the equation of the line, the gradient (slope) of which is equal to the growth rate. All trend lines had an *R*^2^ value of at least 0.99. The apparent lag phase was measured as the time taken for the first OD doubling (determined as an OD of 0.2). The final OD at 600 nm (OD_600_) corresponded to the highest optical density reached by cultures during growth.

### Exposure to antimicrobials.

M. tuberculosis from the logarithmic-growth stage was inoculated into 10 ml of supplemented 7H9 medium containing an antimicrobial agent and was incubated at 37°C with shaking for 3 days. The initial inocula in all experiments were (4 ± 1.5) × 10^7^ CFU/ml. After centrifugation at 4,000 × *g*, pellets were washed once with 7H9 medium, resuspended in 1 ml of supplemented 7H9 medium, and used for the estimation of CFU, MPN, and MNP counts in the presence of culture supernatant (MPN_SN). Antimicrobial concentrations (in micrograms per milliliter) were as follows: streptomycin, 20; isoniazid, 10; ethambutol, 20; rifampin, 5. The exposure of M. tuberculosis to these drug concentrations resulted in marked decreases in CFU counts (>2 log_10_).

M. smegmatis was inoculated from a single colony into 5 ml of LB supplemented with 0.05% (wt/vol) Tween 80 and was grown at 37°C with shaking at 200 rpm for 24 h. The culture (1 ml) was then centrifuged for 10 min at 14,000 × *g* and was resuspended in 1 ml of Sauton's medium supplemented with one of the following drugs (with concentrations given in micrograms per milliliter): amikacin (100), meropenem (50), rifampin (5, 25, or 100), ethambutol (20), isoniazid (1, 10, or 100), or streptomycin (10 or 20). Samples were incubated at 37°C with shaking for 24 h. After washing with Sauton's medium, the treated cells were resuspended in 1 ml of Sauton's medium and were used for CFU, MPN, and MPN_SN assays. When strains containing pMind plasmids were assessed, kanamycin was added at a final concentration of 50 μg/ml at all stages of the experiment. Plates were incubated for 2 weeks statically at 37°C before the number of positive wells was recorded. Each sample was analyzed in triplicate; duplicate biological replicas were used per assay; and each independent assay was performed at least twice

### Generation of Rpf-overexpressing strains.

The *rpfABCDE* genes were amplified from the M. tuberculosis genome by PCR using the gene-specific primers listed in Table S1 in the supplemental material. The PCR products were cloned into the BamHI and MluI (24) sites of the pMind plasmid or pMind-6×His-Myc (23), and the constructs were sequenced by GATC Biotech (Germany) before electroporation into M. smegmatis. The transformants were selected by incorporating kanamycin at a final concentration of 50 μg/ml into the growth medium. The identities of the resultant strains were confirmed using colony PCR and the pMind-specific primers MindF and MindR. A control M. smegmatis strain contained an empty pMind plasmid. *rpf* expression was induced by the addition of 20 ng/ml tetracycline. The plasmids and strains generated in this study are shown in Table 1. The MICs of rifampin and isoniazid for pMind and the RpfD-overexpressing strain were determined by the microdilution method as described previously (23).

**TABLE 1:** Strains generated in this study

Plasmid name	Strain	Insert size (bp)^*a*^	Primers	Description
pMind::*rpfA*	RpfA overexpression	1,224	RpfAF and RpfAR	M. tuberculosis *rpfA* gene cloned into pMind
pMind::*rpfB*	RpfB overexpression	1,089	RpfBF and RpfBR	M. tuberculosis *rpfB* gene cloned into pMind
pMind::*rpfC*	RpfC overexpression	531	RpfCF and RpfCR	M. tuberculosis *rpfC* gene cloned into pMind
pMind::*rpfD*	RpfD overexpression	465	RpfDF and RpfDR	M. tuberculosis *rpfD* gene cloned into pMind
pMind::*rpfE*	RpfE overexpression	519	RpfEF and RpfER	M. tuberculosis *rpfE* gene cloned into pMind
pMind::*rpf*	Rpf overexpression	696	RpfF and RpfR	M. luteus rpf gene cloned into pMind
pMind	MIND	N/A	N/A	Empty plasmid control

a N/A, not applicable.

### RNA extraction and qRT-PCR.

RNA was extracted from 10 ml of mid-log-phase mycobacterial cultures using a previously described TRIzol method (25). To remove DNA contamination, RNA samples were cleaned using the Turbo DNA-*free* kit (Invitrogen). cDNA synthesis, using the commercially available SuperScript II reverse transcriptase kit (Invitrogen), was performed according to the manufacturer's instructions. Quantitative real-time PCR (qRT-PCR) experiments were carried out in a Corbett Rotor Gene 6000 real-time thermocycler. Absolute qPCR SYBR green mix (ABgene) and gene-specific primers were used as described previously (26). The level of expression of each individual M. tuberculosis rpf gene was normalized to that of 16S rRNA (26). Amplification efficiencies were automatically calculated using Rotor Gene 6000 series software. Melting curves were used to confirm the specificity of the qPCR product.

### Detection of Rpf in culture supernatants.

Enzyme-linked immunosorbent assays (ELISAs) were carried out using a method adapted from previously published work (15). Briefly, supernatants (10 ml) were obtained from logarithmic-phase cultures grown in Sauton's medium. After centrifugation and filtration, supernatants were concentrated by freeze-drying. Dried samples were resuspended in 1 ml phosphate-buffered saline (PBS). ELISA was performed in 96-well plates with 100 μl of a concentrated culture supernatant. Primary monoclonal anti-polyhistidine (Sigma-Aldrich) or polyclonal anti-Rpf (13) antibodies and alkaline phosphatase-conjugated secondary anti-mouse or anti-sheep antibodies (respectively) were used at dilutions of 1:3,000 and 1:10,000, respectively. A *p*-nitrophenyl phosphate substrate (Sigma-Aldrich) was used for the detection of alkaline phosphatase activity. The reaction was measured spectrophotometrically at 405 nm, using a Model 680 microplate reader (Bio-Rad).

## RESULTS

### Drug treatment induces culture supernatant dependency in mycobacteria.

**FIG 1:**
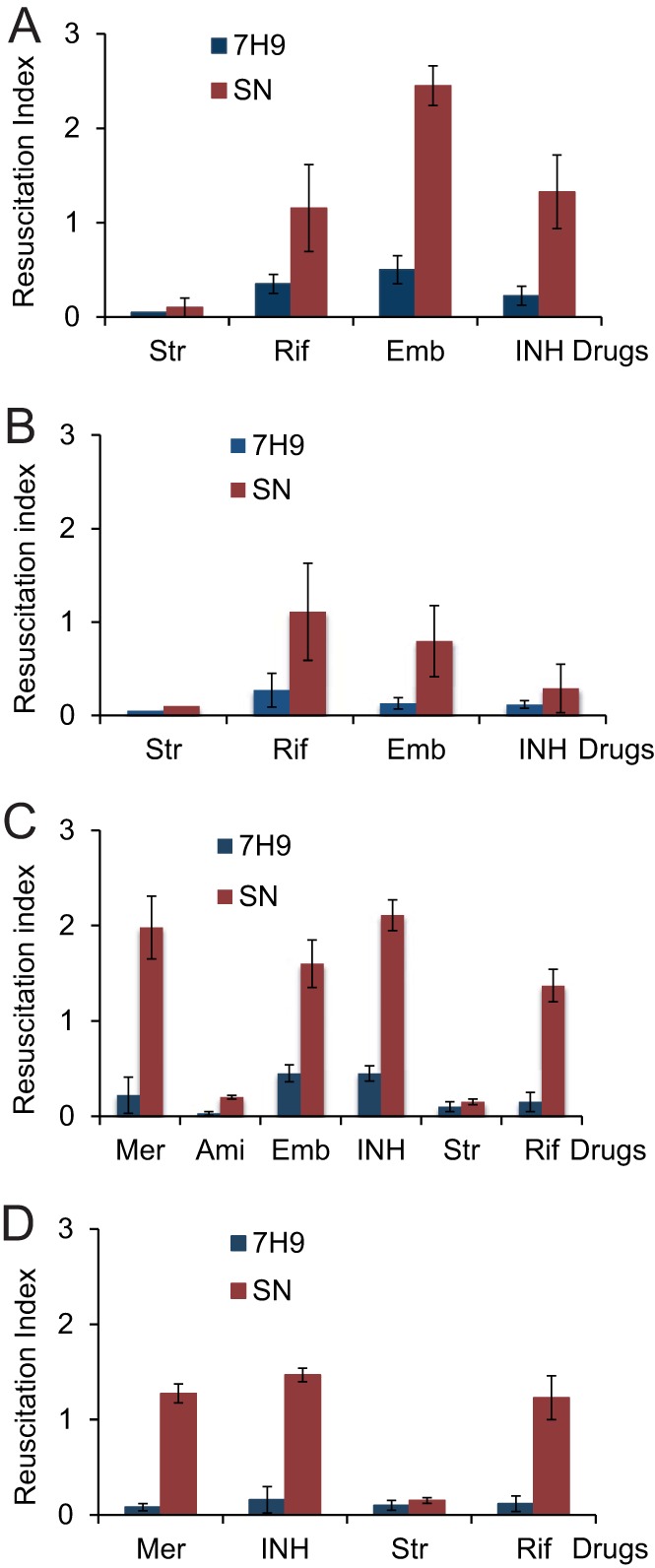
Antimicrobial treatment induces SN dependency in mycobacteria. M. tuberculosis (A and B) or M. smegmatis (C and D) was treated with antimicrobial agents as described in Materials and Methods. M. tuberculosis was exposed to drugs for 3 (A) or 7 (B) days. M. smegmatis was treated for 24 h (C) or 48 h (D). The resuscitation index was calculated as log_10_(MPN) − log_10_(CFU). Str, streptomycin; Rif, rifampin; Emb, ethambutol; INH, isoniazid; Mer, meropenem; Ami, amikacin. Average values for two independent experiments with three biological replicates are shown; error bars indicate standard deviations.

Previous studies have shown that antimicrobial treatment of mycobacteria can result in the generation of metabolically distinct forms (12). Thus, the presence of NP mycobacteria in the drug-treated population was investigated. M. tuberculosis cultures were treated with isoniazid, rifampin, ethambutol, or streptomycin for 3 days, and viable counts of surviving cells were determined using CFU and MPN counts with or without the addition of Rpf-containing culture supernatants (Rpf-SN) (see Fig. S1 in the supplemental material). As shown in Fig. 1A and Fig. S1, no significant resuscitation was observed in 7H9 medium after treatment with any of these drugs. However, the addition of Rpf-SN caused significant increases in viable counts after treatment with ethambutol, isoniazid, or rifampin. Interestingly, streptomycin was very efficient at eliminating mycobacteria and did not result in the generation of resuscitatable mycobacteria. Treatment of mycobacteria with 10 or 20 μg/ml of streptomycin resulted in similar outcomes (data not shown). Further incubation of mycobacteria with drugs led to reductions in resuscitation indices for isoniazid and ethambutol but not for rifampin (Fig. 1B; also Fig. S1), showing that cell wall-targeting antimicrobials induce temporary SN dependency.

Next, the propensity of drugs to induce SN dependency was investigated using M. smegmatis cultures (Fig. 1C and D; see also Fig. S2 in the supplemental material). While streptomycin and amikacin did not induce SN dependency, meropenem, ethambutol, isoniazid, and rifampin generated high numbers of mycobacteria that could be resuscitated only in the presence of a culture supernatant. However, as with M. tuberculosis culture, further incubation with drugs reduced the numbers of resuscitating mycobacteria in samples treated with meropenem or isoniazid (Fig. 1D). These data indicate that certain drugs can result in the generation of NP mycobacteria, which can be detected by the application of a liquid culture supplemented with Rpf-containing culture supernatants. To simplify this assay and prove the resuscitating effect of Rpfs, we generated M. smegmatis strains that artificially overproduce M. tuberculosis Rpfs and validated their usefulness for the detection of Rpf-dependent mycobacteria induced by drug treatment.

### Generation and validation of strains overexpressing *rpf* genes.

**FIG 2:**
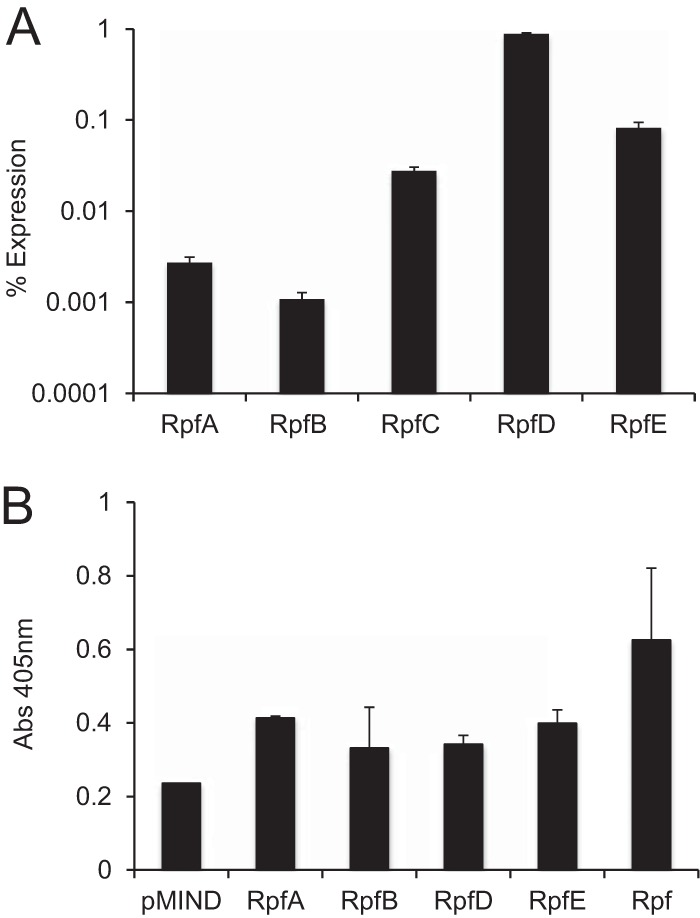
Confirmation of M. tuberculosis rpf gene overexpression and Rpf production in M. smegmatis strains. (A) Expression of *rpfA* through *rpfE* as a percentage of 16S rRNA expression. (B) Detection of RpfA, RpfB, RpfD, and RpfE in M. smegmatis culture supernatants by ELISA with monoclonal anti-polyhistidine antibodies. A recombinant His-tagged form of RpfB (designated Rpf) was used as a positive control. RpfC production was not assessed.

Each of the five M. tuberculosis *rpf* genes (*rpfABCDE*) was cloned into the pMind or pMind-6×His-Myc plasmid, and the level of *rpf* expression was assessed using qRT-PCR. In separate experiments, the amounts of Rpfs released into culture supernatants were determined by ELISAs using anti-polyhistidine or anti-Rpf (13) antibodies. As can be observed in Fig. 2A, the M. tuberculosis *rpf* genes were expressed from the pMind plasmid at different levels: *rpfD* was expressed at the highest level, followed by *rpfE*, *rpfC*, *rpfA*, and *rpfB*. Measurement of Rpfs in culture supernatants using polyclonal anti-polyhistidine antibodies confirmed the increases in Rpf levels in the overexpressing strains: those overexpressing RpfA and RpfE showed the greatest absorbance, followed by those with RpfB and RpfD (Fig. 2B). Similar results were obtained with anti-Rpf antibodies (data not shown). The apparent discrepancy between the qRT-PCR data and the ELISA results could be explained by the attachment of Rpfs to the cell wall, as demonstrated previously (27).

Next, the effects of M. tuberculosis *rpf* overexpression on the growth of M. smegmatis were examined. Growth experiments were carried out in supplemented 7H9 medium, and growth parameters were calculated (Table 2). The overexpression of any M. tuberculosis *rpf* gene resulted in a slight but statistically significant increase in the final OD over that for the control strain (*P*, <0.05 by the *t* test). Differences in the growth rate were also observed; RpfA, RpfB, and RpfC led to increases in the growth rate, while RpfE led to a slightly reduced growth rate. RpfD and Micrococcus luteus Rpf led to growth rates comparable to that of the control strain. Examination of the apparent lag phase revealed a slight reduction in strains overexpressing *rpfC* or *rpfD* and a smaller reduction in strains overexpressing *rpfE* or M. luteus *rpf* (Table 2). Overexpression of M. tuberculosis Rpfs also altered the growth of M. smegmatis in Sauton's broth (see Fig. S3 in the supplemental material).

**TABLE 2:** Comparative analysis of growth parameters of strains overexpressing *rpf* genes

Strain or description	Apparent lag phase (h)^*a*^	Final OD^*b*^	Growth rate (h^−1^)^*b*^^,^^*c*^
MIND	20 ± 0.66	0.71	0.19
RpfA overexpression	20 ± 0.88	0.82*	0.22*
RpfB overexpression	20 ± 0.71	0.82*	0.21*
RpfC overexpression	18 ± 0.88	0.81*	0.20*
RpfD overexpression	18 ± 0.66	0.81*	0.19
RpfE overexpression	19 ± 0.5	0.81*	0.18*
M. luteus Rpf overexpression	19 ± 0.53	0.84*	0.19

a Values are means ± standard deviations.

b Asterisks indicate values significantly different from that for the control strain, MIND (*P*, <0.05 by the *t* test).

c Growth parameters were calculated as described in Materials and Methods.

**FIG 3:**
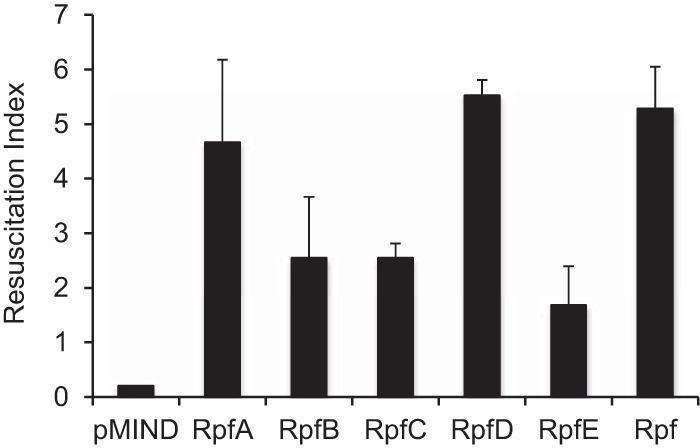
Overexpressed M. tuberculosis rpf genes resuscitate nonplatable M. smegmatis. NP mycobacteria were generated in potassium-limited medium. Resuscitation was carried out in Sauton's medium supplemented with 50 μg/ml of kanamycin and 20 ng/ml of tetracycline. The resuscitation index was calculated as log_10_(MPN) − log_10_(CFU). Average values for three independent experiments are shown; error bars indicate standard deviations. The limit of detection was 0.2. The limit-of-detection value is shown for the MIND strain.

Rpfs have previously been implicated in the resuscitation of NP M. tuberculosis cells and have shown cross-species reactivity, including reactivity in M. smegmatis and Mycobacterium bovis BCG (13, 14). The resuscitation abilities of the individual M. tuberculosis Rpfs in the M. smegmatis Rpf-overexpressing strains were established in a control system prior to assessment of the front-line antituberculosis drugs. To determine the resuscitation capabilities of *rpf*-overproducing strains, we used a well-established M. smegmatis NP model (15). This assay uses potassium-limiting conditions to generate NP cells that are unable to grow in standard media and can be resuscitated only by the addition of SN or recombinant Rpf. All five strains overexpressing M. tuberculosis Rpfs produced no colonies on agar but resuscitated in liquid medium. In agreement with the data published previously, M. smegmatis overexpressing M. luteus Rpf (15) was also able to grow in liquid culture, while the empty-plasmid control failed to resuscitate (Fig. 3). RpfA and RpfD had the greatest resuscitation capacity, as reflected by the highest resuscitation index values (4.7 and 5.3, respectively), followed by RpfB, RpfC, and RpfE. Interestingly, *rpfD* was the most overexpressed gene by qRT-PCR, while RpfA was the most abundant Rpf protein detected in culture supernatants by ELISA. These results indicated that Rpf-overexpressing strains could be used for the detection of Rpf-dependent mycobacteria.

### Rpf overexpression rescues nonculturable mycobacteria generated by treatment with front-line drugs.

**FIG 4:**
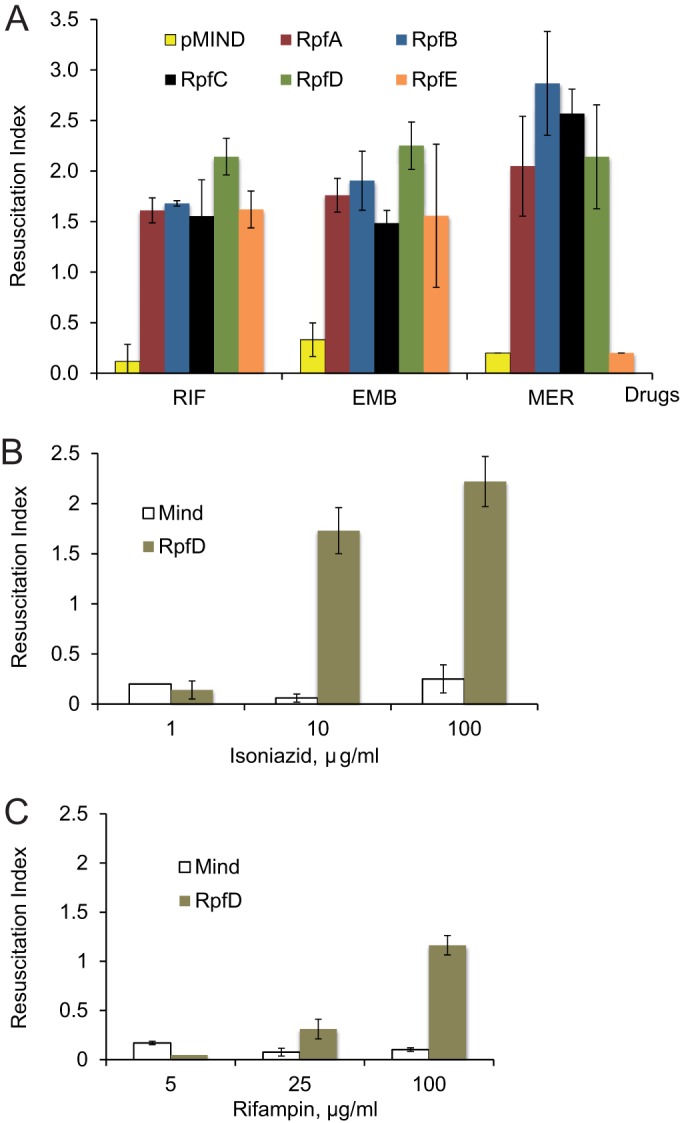
Use of strains overexpressing *rpf* genes for the detection of Rpf-dependent mycobacteria induced by treatment with antituberculosis drugs. Strains were treated with ethambutol (EMB), meropenem (MER), or rifampin (RIF) (A), isoniazid (B), or rifampin (C). The resuscitation index was calculated as log_10_(MPN) − log_10_(CFU). Average values for three independent experiments are shown; error bars indicate standard deviations. The limit of detection was 0.2.

To investigate the abilities of individual Rpfs to resuscitate drug-treated mycobacteria, M. smegmatis Rpf-overexpressing strains and the control strain MIND were treated with rifampin, ethambutol, or meropenem, and the numbers of surviving bacteria were determined by CFU and MPN counts. CFU counts in all treated populations decreased by ∼2 orders of magnitude from those with the initial inocula (see Fig. S4 in the supplemental material). Figure 4A shows resuscitation indices calculated for rifampin-, meropenem-, and ethambutol-treated samples. The highest values were obtained for meropenem-treated samples, confirming the data for M. smegmatis presented in Fig. 1C. Rifampin and ethambutol treatment also led to the generation of Rpf-dependent mycobacteria. On the other hand, CFU counts for streptomycin-treated samples were below the limit of detection, corresponding to 25 cells/ml, and no resuscitation was observed for any of the Rpf overexpression strains, suggesting that this drug treatment does not induce Rpf dependency (data not shown). Reducing the streptomycin concentration to 10 μg/ml did not increase viable counts. This apparently increased sensitivity of Rpf overexpression strains to streptomycin may be caused by the presence of the plasmids or the administration of kanamycin for their maintenance, or both.

Among the strains overexpressing different Rpfs, the RpfD-overexpressing strain showed consistently higher resuscitation than the others, while the RpfE-overexpressing strain showed the lowest resuscitation potential. In accordance with previously published data, the empty-plasmid strain did not resuscitate in liquid medium, while the positive-control strain overexpressing M. luteus Rpf showed high resuscitation potential (15).

We next investigated how different concentrations of drug influence the generation of Rpf-dependent mycobacteria. We treated the pMind control strain and the RpfD-overexpressing strain with three different concentrations of isoniazid (1, 10, and 100 μg/ml). According to our data, the isoniazid MICs for the pMind and RpfD-overexpressing strains were identical (5 μg/ml). As Fig. S5 in the supplemental material shows, at a concentration of 1 μg/ml, isoniazid did not influence the viability of M. smegmatis and did not result in the generation of RpfD-dependent mycobacteria, while high numbers of RpfD-dependent mycobacteria arose after exposure to 10 or 100 μg/ml of isoniazid (Fig. 4B). A similar pattern was obtained with rifampin-treated mycobacteria. The rifampin MIC was 16 μg/ml for both strains. At 5 μg/ml rifampin, mycobacteria were able to grow without a loss of viability and did not produce RpfD-dependent forms (Fig. 4C; also Fig. S5). Treatment with 25 μg/ml rifampin stopped the growth of mycobacteria but did not stimulate RpfD dependency; exposure to 100 μg/ml of rifampin resulted in the accumulation of RpfD-dependent M. smegmatis cells. An increase in the drug exposure time to 48 h did not significantly change the viable counts (data not shown); we noted outgrowth of mycobacteria if the time of incubation was extended beyond 72 h. The observed outgrowth of mycobacteria could be caused by the multiplication of drug-resistant mutants or the degradation of drugs.

## DISCUSSION

Tuberculosis is a worldwide health threat, and recent global efforts have been directed to the development of novel agents to eradicate M. tuberculosis and especially its nonreplicating forms, which are believed to be responsible for prolonged treatment and disease relapse. Populations of nonreplicating, Rpf-dependent, and SN-dependent cells were recently identified in patient sputum samples and were apparently enriched in treated samples (16, 28), leading to our hypothesis that antituberculosis drugs may induce Rpf dependency. Accordingly, we investigated whether mycobacteria from growing cultures could become NP or SN dependent after exposure to antimicrobial agents. In our experiments, we did not detect substantial numbers of NP mycobacteria that could be regrown in liquid medium (Fig. 1). However, mycobacterial populations treated with either rifampin, ethambutol, isoniazid, or meropenem did indeed develop SN dependency. This phenomenon was observed for M. tuberculosis and M. smegmatis, suggesting that it is not associated with slow-growing mycobacteria. The numbers of SN-dependent mycobacteria obtained after treatment with cell wall-targeting drugs decreased after extended incubation (Fig. 1B and D), though rifampin-induced SN-dependent populations remained stable even during prolonged treatment (Fig. 1B and D). We have shown previously that the resuscitation effect of culture supernatant was caused mainly by Rpf proteins (11, 14, 16). Therefore, we hypothesized that artificial overexpression of Rpf in treated mycobacteria might induce their resuscitation, and consequently, we developed a model system using M. smegmatis strains overexpressing M. tuberculosis Rpfs.

The expression of each M. tuberculosis rpf gene was confirmed at the RNA level by qRT-PCR (Fig. 2A); the release of Rpf proteins was confirmed by ELISA (Fig. 2B). The Rpf-overexpressing strains showed a growth advantage in 7H9 medium (Table 2) and in Sauton's broth (Fig. S3 in the supplemental material); they were also able to resuscitate in an NP model (Fig. 3), suggesting their potential utility for the investigation of drug-induced Rpf-dependent mycobacteria. Our results indicate that overexpressed Rpfs resuscitate Rpf-dependent M. smegmatis (Fig. 4). Moreover, the resuscitation effects of overexpressed Rpfs were very similar to those of culture supernatant (Fig. 1B).

While the biological significance of these Rpf-dependent populations awaits further evaluation, our findings indicate that the bactericidal actions of meropenem, isoniazid, ethambutol, and rifampin may have been overestimated. Interestingly, neither streptomycin nor amikacin induced Rpf-dependent mycobacteria in any of our assays, suggesting that aminoglycosides are potent drugs for the prevention of Rpf dependency in mycobacteria. The Rpf proteins are the enzymes involved in the remodeling, biosynthesis, and (potentially) repair of bacterial cell walls. Any environmental insult, including drug treatment, leading to alteration or damage of the cell wall may put bacteria in a situation where the role of cell wall-modifying enzymes, such as Rpfs, becomes vital. Thus, the data presented indicate that drug-induced Rpf dependency may be associated with inhibition or damage of cell wall biosynthesis but not with a shutdown of protein biosynthesis. We also cannot exclude the possibility that drug treatment helps to reveal some “preexisting” Rpf-dependent populations by eliminating most of the platable bacteria from actively growing cultures. The phenomenon of “persisters” has long been recognized, and persisters are believed to arise stochastically in growing cultures (29).

We have demonstrated recently that SN-dependent mycobacteria from sputum are more tolerant to isoniazid and streptomycin than actively growing mycobacteria (17). This potentially means that Rpf-dependent M. tuberculosis bacilli generated *in vivo* are more resistant to protein biosynthesis inhibition than bacteria from actively growing cultures and that the enrichment of SN- or Rpf-dependent M. tuberculosis bacilli in samples from treated patients is due to their higher tolerance to drugs. Interestingly, treatment of growing cultures with ethambutol induced SN dependency both in M. tuberculosis and in M. smegmatis; however, SN-dependent mycobacteria from sputum did not show higher tolerance to this drug than actively growing mycobacteria (17), suggesting the complex nature of the SN dependency phenomenon. Nevertheless, it is possible that some Rpf-dependent M. tuberculosis bacilli isolated from posttreatment samples resulted from drug exposure. Thus, our M. smegmatis model system can be used for the detection of drugs with a high propensity to induce Rpf dependency. Our assay using Rpf-overexpressing strains in the nonpathogenic bacterium M. smegmatis has been shown to accurately model the effects observed in M. tuberculosis. Drug resistance in TB is on the rise, and current efforts are focused on developing new antibiotics that will be effective against multidrug-resistant TB (MDR-TB) and extensively drug resistant TB (XDR-TB). It is therefore worthwhile to investigate a simple *in vitro* method for assessing the Rpf-dependent populations produced by antimicrobial treatments in order to help prevent resistance in the future.

## Supplementary Material

Supplemental material
