# Supplementary material for: Development of an In Vitro Assay for Detection of Drug-Induced Resuscitation-Promoting-Factor-Dependent Mycobacteria
Source: Antimicrob Agents Chemother. 2016 Sep 23;60(10):6227–33. doi: 10.1128/AAC.00518-16 (PMC5038329; doi:10.1128/AAC.00518-16)
Supplement: Supplemental material [file supp_60_10_6227__index.html]

Development of an In Vitro Assay for Detection of Drug-Induced Resuscitation-Promoting-Factor-Dependent Mycobacteria — Supplemental material 

# Development of an *In Vitro* Assay for Detection of Drug-Induced Resuscitation-Promoting-Factor-Dependent Mycobacteria

## Supplemental material

- Supplemental file 1 -

  Tables S1 and S2 and Figures S1 to S5

  PDF, 808K
