# Supplementary material for: Development of an In Vitro Assay for Detection of Drug-Induced Resuscitation-Promoting-Factor-Dependent Mycobacteria
Source: Antimicrob Agents Chemother. 2016 Sep 23;60(10):6227–33. doi: 10.1128/AAC.00518-16 (PMC5038329; doi:10.1128/AAC.00518-16)
Supplement: Supplemental material [file AAC.00518-16_zac010165595so1.pdf]

Development of an *in vitro* assay for detection of Resuscitation-promoting factor dependent mycobacteria induced by treatment with antimicrobial agents

Jessica Loraine[$], Feifei Pu[&], Obolbek Turapov, Galina V. Mukamolova

Department of Infection, Immunity and Inflammation, University of Leicester, Leicester, UK

[$] Current address: UCL School of Pharmacy, University College London, London, UK

[&] Current address: Department of Clinical Training Center Diagnostics Division, Siemens China, Shanghai, China

*To whom correspondence should be addressed. E-mail: gvm4@le.ac.uk

Running Title: Drug-induced Rpf-dependency

Content

Table S1. Primers used in the study.

Table S2. Viable counts for mycobacterial cultures prior to drug exposure

Figure S1. Effect of drug treatment on viability of *M. tuberculosis.*

Figure S2. Effect of drug treatment on viability of *M. smegmatis*

Figure S3. Effect of Rpf over-expression on *Msm* growth in Sauton's medium.

Figure S4. Viable counts of untreated, rifampin- and ethambutol- treated *M.smegmatis*

Figure S5. Viable counts of *M. smegmatis* strains exposed to different concentrations of isoniazid and rifampin.

Table S1. Primers used in the study

Primer	Sequence 5’-3’	Description
RpfAF	AGTGGATCCATGTTGCGCCTGGTAGTCGGTGCG	*Mtb rpfA* cloning in pMind
RpfAR	ATAACTAGTTCAACGCGTGCGCGCACCCGCTCGTGCAGC	*Mtb rpfA* cloning in pMind
RpfBF	AGTGGATCCATGTTGCGCCTGGTAGTCGGTGCG	*Mtb rpfB* cloning in pMind
RpfBR	ATAACTAGTTCAACGCGTGCGCGCACCCGCTCGTGCAGC	*Mtb rpfB* cloning in pMind
RpfCF	AGTGGATCCGTGCATCCTTTGCCGGCCGACCAC	*Mtb rpfC* cloning in pMind
RpfCR	TATACTAGTTCACATATGGCGCGGAATACTTGCCTGAAT	*Mtb rpfC* cloning in pMind
RpfDF	AGTGGATCCCAGCAAGGTGGAGCTGCTATG	*Mtb rpfD* cloning in pMind
RpfDR	CATACTAGTTCAACGCGTATCGTCCCTGCTCCCCGAACA	*Mtb rpfD* cloning in pMind
RpfEF	TCGGGATCCGCGAAAGGAACAACGTTGAAGAAC	*Mtb rpfE* cloning in pMind
RpfER	TGCACTAGTTCACGCGTGCCGCGGCGGCCGCAG	*Mtb rpfE* cloning in pMind
RpfF	TGCCGGATCCGCCGATCAGCGAGGA	*rpf* cloning in pMind
RpfR	GTCACTAGTTCAGGCCTGCGGCAG	*rpf* cloning in pMind
RT-RpfAF	CGG*ACTAGT*CTA GCCAACGATGATGAT	qRT-PCR *Mtb rpfA*
RT-RpfAR	TCAGAACGGAATCATCCACCGTGA	qRT-PCR *Mtb rpfA*
RT-RpfBF	GCGATGCCGAAATCCATCACCTTT	qRT-PCR *Mtb rpfB*

RT-RpfBR	AGAACCTCAACGTCTACGGCTTCA	qRT-PCR *Mtb rpfB*
RT-RpfCF	AGCTGCCTCTCGGGAACAA	qRT-PCR *Mtb rpfC*
RT-RpfCR	GACCACAGTGCGATCGGAAGG	qRT-PCR *Mtb rpfC*
RT-RpfDF	GCAACAGATCGAGGTCGCAG	qRT-PCR *Mtb rpfD*
RT-RpfDR	CGAGGAACGTCAGGATGTGG	qRT-PCR *Mtb rpfD*
RT-RpfEF	T GGCCTACAGCGTGAACTGG	qRT-PCR *Mtb rpfE*
RT-RpfER	GAACGCAGCACGTTCTCCAGC	qRT-PCR *Mtb rpfE*
16srRNAF	TCCGGGCCTTGTACACA	qRT-PCR *16s rRNA*
16srRNAR	TAACACCCGAAGCCAGTGG	qRT-PCR *16s rRNA*
MindF	TGAGTCATAGTTGCACTTTATCAT	Primer for pMind
MindR	TCCGAATCAATACGGTCTAGAGA	Primer for pMind

*Restriction sites are outlined

Table S2. Viable counts for mycobacterial cultures prior to drug exposure

Strain	Log10 CFU ±STDV	Log10 MPN ±STDV	Log10 MPN_SN ±STDV	Figures for treated samples
M. tuberculosis H37Rv	7.65±0.3	7.68±0.41	7.8	Figure 1 A, B; S1
M. smegmatis	7.54±0.21	7.48±0.27	7.6±0.19	Figure 1 C, D; S2
M. smegmatis pMind	7.42±0.23	7.37±0.21	N/D	Figure 4B, C; S5
M. smegmatis pMind::*rpfD*	7.21±0.03	7.17±0.05	N/D	Figure 4B, C; S5

N/D – not determined

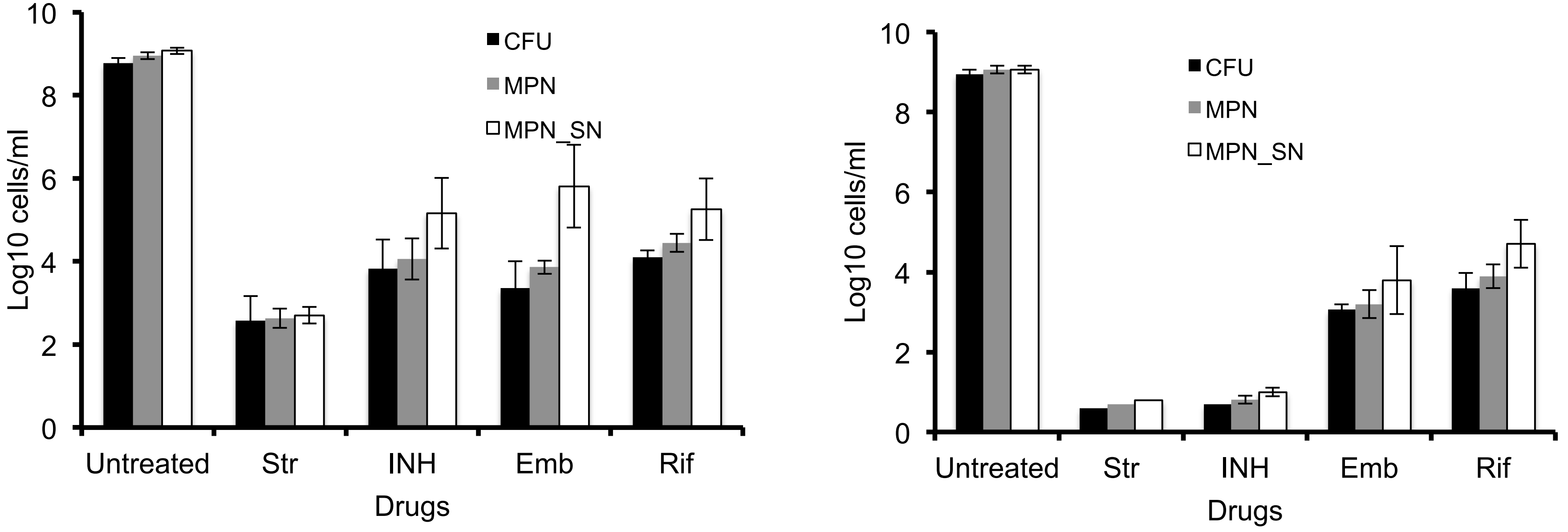

Figure S1. Effect of drug treatment on viability of *M. tuberculosis*. Log phase cultures were inoculated in 7H9 medium without (untreated control) or with drugs and incubated at 37°C with shaking for 3 days (left panel) or 7 days (right panel). Cells were pelleted and washed with 7H9 medium before determination of CFU counts on 7H10 agar or MPN counts in 7H9 medium (MPN) or in 7H9 supplemented with 50% (v/v) culture supernatant. Average of three independent experiments (done in duplicates) are shown. Error bars indicate standard deviations. Drugs were added at the following concentrations (µg/ml): streptomycin (Str) 20, rifampin (RIF) 5, isoniazid (INH) 10, ethambutol (Emb) 20.

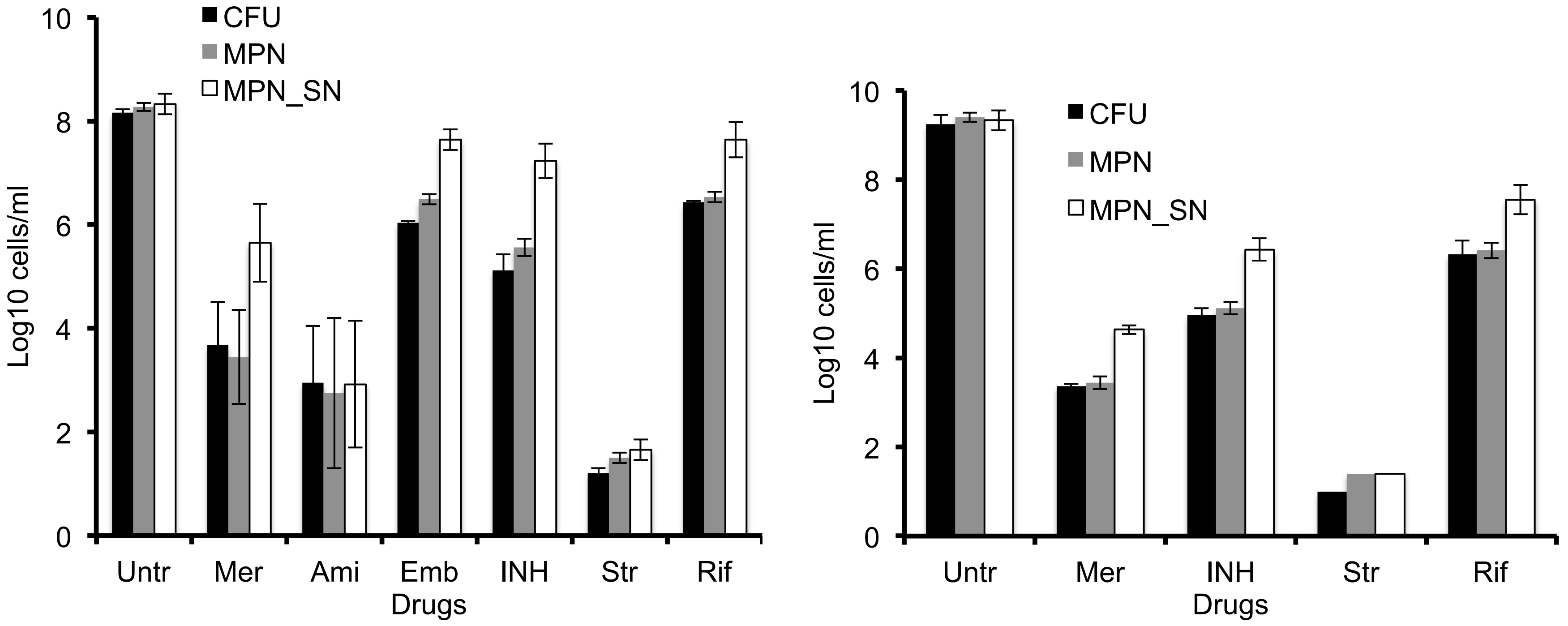

Figure S2. Effect of drug treatment on viability of *M. smegmatis.* Log phase cultures were inoculated in 7H9 medium without (untreated control) or with drugs and incubated at 37°C with shaking for 24 hours (left panel) or 48 hours (right panel). Cells were pelleted and washed with 7H9 medium before determination of CFU counts on 7H10 agar or MPN counts in 7H9 medium (MPN) or in 7H9 supplemented with 50% (v/v) culture supernatant. Average of three independent experiments (done in duplicates) are shown. Error bars indicate standard deviations. Drugs were added at the following concentrations (μg/ml): streptomycin (Str) 20, rifampin (RIF) 100, isoniazid (INH) 10, ethambutol (Emb) 20, meropenem (Mer) 50, amikacin (Ami) 100.

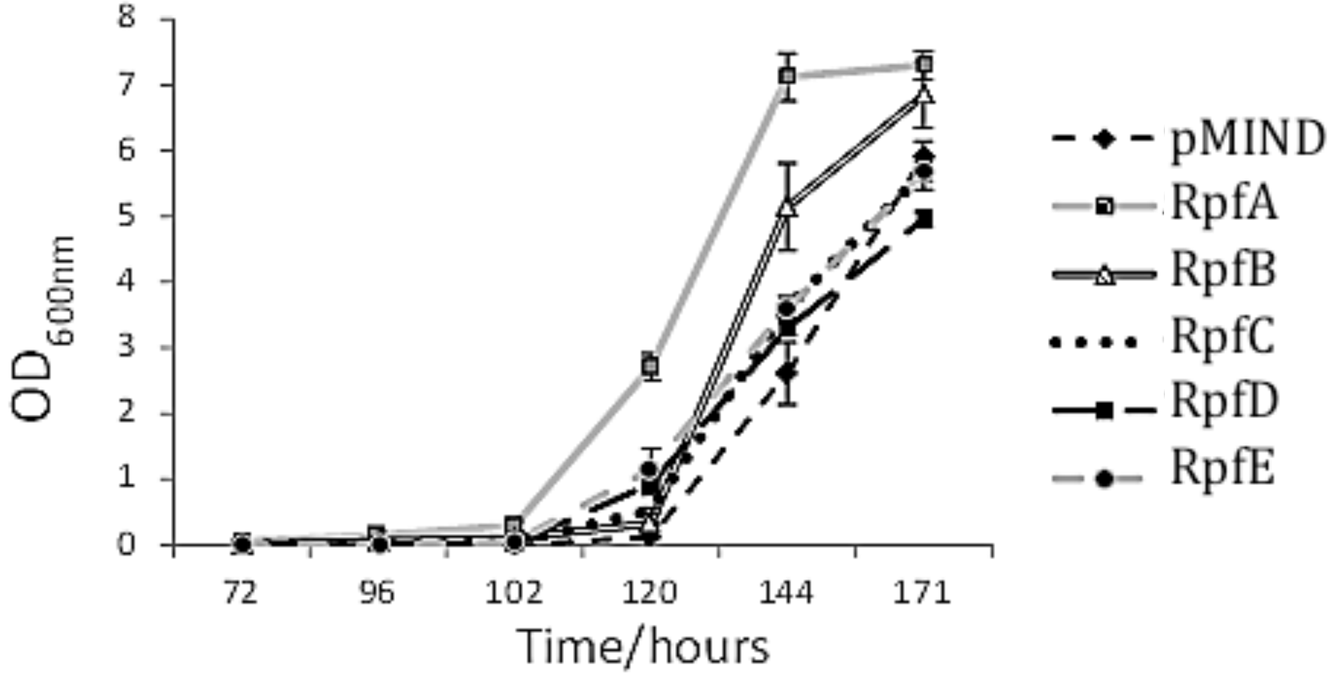

Figure S3. Effect of Rpf overexpression on *M. smegmatis* growth in Sauton's medium. 10 ml *M. smegmatis* cultures were grown in Sauton's medium supplemented with 50 µg/ml kanamycin and 20 ng/ml tetracycline. Average OD_{600} values of three independent experiments are shown, error bars indicate standard deviation.

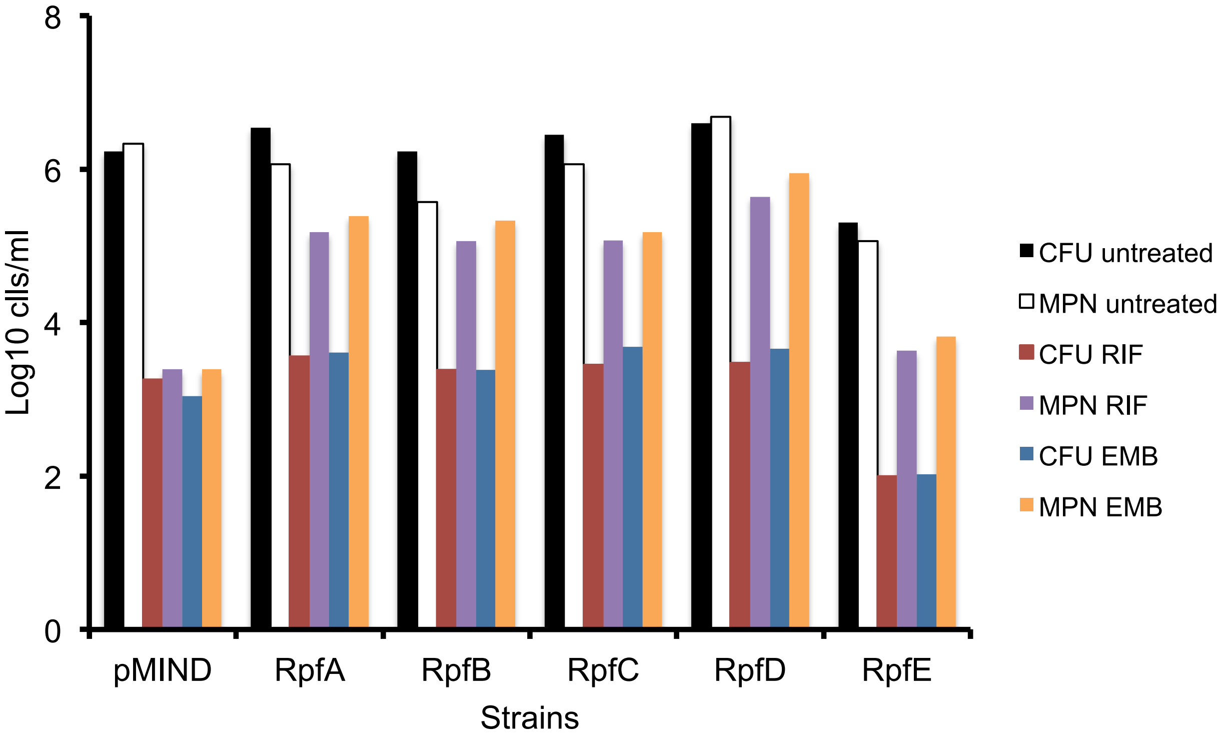

Figure S4. Viable counts of rifampin-, ethambutol-treated and untreated *M.smegmatis*. Mycobacteria from logarithmic growth phase were treated with antimicrobials and their viable counts determined as described in Methods. Drugs were added at the following concentrations (μg/ml): rifampin (RIF) 100 and ethambutol (Emb) 20.

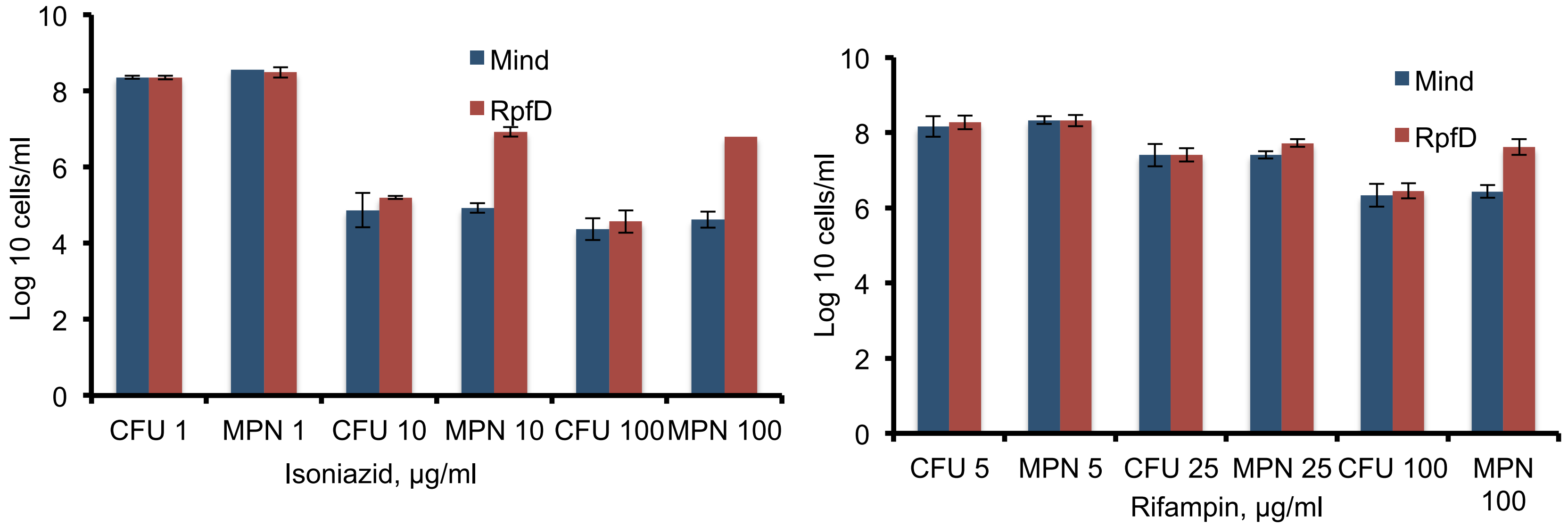

Figure S5. Viable counts of *M. smegmatis* strains exposed to different concentrations of isoniazid (left panel) and rifampin (right panel). Mycobacteria from logarithmic growth phase were treated with antimicrobials for 24 hours and their viable counts determined as described in Methods. Drugs were added at the following concentrations (µg/ml): isoniazid 1, 10 and 100; rifampin 5, 25 and 100.
